# Assessment of ePrescription quality: an observational study at three mail-order pharmacies

**DOI:** 10.1186/1472-6947-9-8

**Published:** 2009-01-26

**Authors:** Bengt Åstrand, Emelie Montelius, Göran Petersson, Anders Ekedahl

**Affiliations:** 1 Apoteket AB, and School of Pure and Applied Natural Sciences, University of Kalmar, Kalmar, Sweden; 2 e-Health Institute, School of Human Sciences, University of Kalmar, Kalmar, Sweden

## Abstract

**Background:**

The introduction of electronic transfer of prescriptions (ETP) or ePrescriptions in ambulatory health care has been suggested to have a positive impact on the prescribing and dispensing processes. Thereby, implying that ePrescribing can improve safety, quality, efficiency, and cost-effectiveness. In December 2007, 68% of all new prescriptions were transferred electronically in Sweden. The aim of the present study was to assess the quality of ePrescriptions by comparing the proportions of ePrescriptions and non-electronic prescriptions necessitating a clarification contact (correction, completion or change) with the prescriber at the time of dispensing.

**Methods:**

A direct observational study was performed at three Swedish mail-order pharmacies which were known to dispense a large proportion of ePrescriptions (38–75%). Data were gathered on all ePrescriptions dispensed at these pharmacies over a three week period in February 2006. All clarification contacts with prescribers were included in the study and were classified and assessed in comparison with all drug prescriptions dispensed at the same pharmacies over the specified period.

**Results:**

Of the 31225 prescriptions dispensed during the study period, clarification contacts were made for 2.0% (147/7532) of new ePrescriptions and 1.2% (79/6833) of new non-electronic prescriptions. This represented a relative risk (RR) of 1.7 (95% CI 1.3–2.2) for new ePrescriptions compared to new non-electronic prescriptions. The increased RR was mainly due to 'Dosage and directions for use', which had an RR of 7.6 (95% CI 2.8–20.4) when compared to other clarification contacts. In all, 89.5% of the suggested pharmacist interventions were accepted by the prescriber, 77.7% (192/247) as suggested and an additional 11.7% (29/247) after a modification during contact with the prescriber.

**Conclusion:**

The increased proportion of prescriptions necessitating a clarification contact for new ePrescriptions compared to new non-electronic prescriptions indicates the need for an increased focus on quality aspects in ePrescribing deployment. ETP technology should be developed towards a two-way communication between the prescriber and the pharmacist with automated checks of missing, inaccurate, or ambiguous information. This would enhance safety and quality for the patient and also improve efficiency and cost-effectiveness within the health care system.

## Background

The introduction of electronic transfer of prescriptions (ETP), or ePrescriptions, in ambulatory health care has been suggested to have a positive impact on the prescribing and dispensing processes implying that ePrescribing can improve safety, quality, efficiency and cost-effectiveness [1-10].

For hundreds of years, the handwritten prescription has been the method of choice for the physician to communicate decisions on drug therapy and for the pharmacist to dispense the medication. At the same time, it has acted as a source of information for the patient about how to use the medication in order to maximize its benefit. Regulation of the written prescriptions used by apothecaries in dispensing drugs appeared in one of the earliest known statutes on the control of drugs [11]. Over the following centuries, both the "recipe" for drug composition and the dispensing of medications at pharmacies became more and more tightly regulated and became subject to inspection by the authorities in order to ensure quality for the patient. Dispensing pharmacists are now obliged to examine all prescriptions for accuracy, completeness and correctness. However, the handwritten prescription has a number of well-recognized weaknesses. Namely, varying readability and interpretation of the prescriber's handwriting, the risk of falsification, unidirectional communication with no feedback and the lack of easily understandable information for the patient. Currently, drug prescribing is at a transitional stage and the adaptation of a traditional process to the new electronic era offers unique challenges.

ETP technology was first deployed in 1983 in an outpatient setting with electronic communication being set up between the computer systems at a doctor's office at the medical clinic and those at a nearby pharmacy in Jönköping, Sweden. The collaboration resulted in the world's first electronically transferred prescription in an outpatient setting [12-14]. In 1984, Swedish authorities regulated the ETP for the first time, including the test package and dose schedule/time period prescribing features [15]. In short, the new regulation made it legal for pharmacists to dispense an electronically transferred prescription with local agreements regulating the responsibility for the sending and the receiving professionals. By making their computer systems available for ePrescriptions, the dispensing pharmacists were made responsible for the quality and safety of ePrescriptions.

Since then, prescription software, including both systems integrated with electronic health care records (EHR) and web-based stand-alone systems, has been developed, marketed and implemented and is currently being introduced on a broad scale within health care. In recent years, in countries such as Sweden, Denmark and the US, considerable efforts have been made towards the wide scale implementation of ePrescribing [4,16-18].

**Figure 1:**
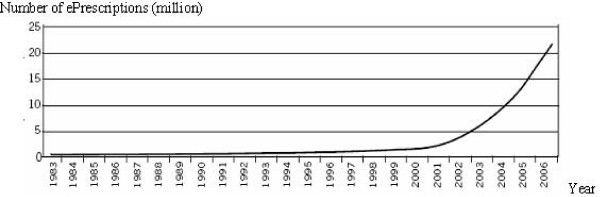
**Trends in transferred ePrescriptions in Sweden where the first ePrescription was launched in 1983 **[16]. In December 2007, 68% (inter-county range 46–85%) of all prescriptions were electronically transferred with a steadily growing trend.

The development of ePrescriptions in Sweden has accelerated rapidly since a new strategy with collaborative national and regional implementation teams was introduced at the end of the 1990s (Figure 1). The new strategy prioritized the implementation of ePrescriptions among all stakeholders in a coordinated way to reach a penetration rate of at least 80%. By the end of 2007, the actual penetration rate of ETP was as high as 68% of all new prescriptions (unpublished observations Apoteket AB, December, 2007). A national ePrescription communication exchange hub with a virtual repository, called the national mail box for prescriptions, allows the patient to access their prescriptions at any pharmacy on presentation of valid identification.

However, the introduction of new technologies such as ePrescribing may create new errors, both systematic and non-systematic, in the prescribing and dispensing processes [19,20]. Stored ePrescription information may be used not only for filling the actual prescription but also for future clinical decision making and epidemiological research. Thus, it is vital to systematically monitor the quality of the electronic prescribing process [21,22].

### Aim of the study

The aim of the present study was to assess the quality of ePrescriptions by comparing the proportions of ePrescriptions and non-electronic prescriptions necessitating a clarification contact (correction, completion or change) with the prescriber at the time of dispensing.

## Methods

### Type of study

The study was a prospective, direct, observational study. Data was collected by trained observers (pharmacy students) using a specifically designed protocol. Ethical approval was not sought for the study.

### Study period

The study was conducted over a three week period (15 weekdays) in February–March 2006 at three mail-order pharmacies (MOP) in Sweden.

### Setting

Four Swedish MOPs were invited to participate and one MOP subsequently declined. The three remaining MOPs were chosen as study settings as they all handle prescriptions for non-pharmacy outlets (about 900) in remote areas. In addition, they serve pharmacy customers directly via mail-order. Non-pharmacy outlets are general food stores and other establishments that are not staffed by pharmacists. They deliver pharmaceuticals in sparsely populated areas as representatives for the pharmacies. The MOPs also had a relatively large proportion (range 38–75%) of ePrescriptions at the time of the study. The same regulations and operating procedures apply to the MOPs as to the other community pharmacies (about 900) in Sweden. However, unlike their colleagues at other pharmacies, pharmacists at the mail-order pharmacies cannot communicate with the patient 'face-to-face' at the pharmacy counter. In addition, since they handle prescriptions from prescribers who may not be in the same neighborhood, they generally do not have personal knowledge of the physicians' prescribing habits. Pharmacies in Sweden do not have access to automated software for prospective drug utilization reviews (pDUR). For example, drug interactions and contraindications [23-25]. All prescriptions in the present study were manually examined by the pharmacists.

### Inclusion and exclusion criteria

The study included prescriptions with prescription errors, ambiguities, or other problems related to drug prescriptions for humans with a Swedish civic number where the pharmacist judged it necessary to make a contact with the prescriber prior to dispensing for clarification, correction, completion, or change. All attempts to contact the prescriber were included in the study regardless of whether they resulted in a contact during the study period. Prescriptions for non-pharmaceuticals (syringes, compression stockings, dressings) or for animals were excluded.

### Definition of ePrescriptions

ePrescriptions were defined as personal prescriptions of drugs for humans electronically transferred from the prescriber's computer system to a national ePrescription mail box which is accessible to all Swedish pharmacies. At the time of the study, ePrescriptions with refills were handled and recorded as ePrescriptions only at the first dispensing occasion. Prescriptions faxed or merely printed on paper by a computer system were not defined as ePrescriptions.

### Work organization

At all three settings, prescriptions were prepared in a two-step process. Prescribing problems detected in the prescription preparation line were transferred to a second line where a pharmacist at a phone desk was available to investigate the problem and make contact with the prescriber if clarification was required. Some of the prescription problems may have been resolved by the pharmacists themselves after consulting the patient or the patient's relatives by phone or by means of other information sources. All contacts with the prescribers were made by the pharmacist at the phone desk. The prescribers were not notified of the study.

### Data collection

The observers closely followed the pharmacists at the phone desk, recording all attempts to make contact with prescribers. A copy of the prescription was attached to the protocol form (See Additional file 1). The observations were recorded and classified according to an internationally developed protocol which was translated into Swedish and modified for the Swedish context [26,27].

### Validation of classification

Examination and supervision of the data classification were performed by one of the authors (AE). Any irregularities in classification were discussed with the observers to achieve consensus and consistency.

### Statistics

The collected data were transferred to electronic form in Microsoft Access^® ^and then exported to Microsoft Excel^® ^for cross tabulation.

The outcome measures (numbers and frequencies of prescriptions necessitating a clarification contact, causes of clarification contacts, time and results of interventions) were related to statistics on dispensed prescriptions obtained from Apoteket AB (personal communication B-M Alsén) for February 2006 for the three pharmacies studied. These statistics were adjusted for number of workdays (15/20) as the study covered a different time period (15 workdays) to the collected statistics (20 workdays). Relative risks (RRs) with 95% confidence intervals (95% CIs) were used to compare rates between the two groups ePrescriptions and non-ePrescriptions. RRs and 95% CIs were calculated using Episheet http://ken.rothman.name.

## Results

The statistics for dispensed drug prescriptions during the month of February 2006 (adjusted for 15/20 workdays) comprised 41634 (adjusted to 31225) prescriptions. 46.0% consisted of new prescriptions (inter-pharmacy range 42.8–50.0%). Of these new prescriptions, 52.4% (inter-pharmacy range 38.0–74.6%) were transferred as ePrescriptions (Table 1).

**Table 1:** Number (percentage) of prescriptions dispensed during the study period (three weeks in February 2006).

	All prescriptions N = 31225	Refill prescriptions N = 16860 (54.0%)	All new prescriptions N = 14365 (46.0%)	
			**New ePrescriptions **N = 7532 (52.4%)	**New non-electronic Prescriptions **N = 6833 (47.6%)
			
Pharmacy 1	5880	3127 (53.2%)	2055 (74.6%)	698 (25.3%)
Pharmacy 2	10472	5232 (50.0%)	3058 (58.4%)	2182 (41.6%)
Pharmacy 3	14873	8501 (57.2%)	2419 (38.0%)	3953 (62.0%)

Only ordinary drug prescriptions dispensed for humans are included. Monthly statistics are adjusted (15/20) for the three week period.

Source: Apoteket AB Sweden

Clarification contacts were made for 2.0% (147/7532) of new ePrescriptions and 1.2% (79/6833) of new non-electronic prescriptions. This resulted in an RR of 1.7 (95% CI 1.3–2.2) for new ePrescriptions compared to new non-electronic prescriptions. The increased RR for ePrescriptions was mainly due to one particular cause of intervention. Namely, the 'Dosage and directions for use'. The RR for this cause compared to other causes of intervention was 7.6 (95% CI 2.8–20.4) (Table 2).

**Table 2:** Causes of clarification contacts, presented according to type of prescription, in three mail-order pharmacies over a three week period.

	All prescriptions (N = 31225)	Refill prescriptions (N = 16860)	New ePrescriptions (N = 7532)	New non-electronic prescriptions (N = 6833)	Relative risk (95% CI)
Number of prescriptions necessitating a clarification contact	312	86	147	79	1.7 (1.3–2.2)
Total number of causes of clarification contacts	348	103	150	95	-
**Causes**					
					
**Incorrect or incomplete prescribing**					
Drug, strength, dosage form	45	10	15	20	0.5 (0.3–0.9)
Dosage and directions for use	73	21	48	4	7.6 (2.8–20.4)
Quantity or duration of therapy	21	5	8	8	0.6 (0.3–1.6)
Other	39	8	14	17	0.5 (0.3–1.0)
Prescriber, patient, or discount information	23	2	7	14	0.3 (0.1–0.8)
**Interactions**					
Drug-drug	6	3	2	1	1.3 (0.1–13.8)
**Other causes**					
Adverse effects, toxicity, illegible prescription, falsified prescription, patients' concerns, missing date, or other	44	16	17	11	1.0 (0.5–2.0)
Drug temporarily unavailable or withdrawn from the market	97	38	39	20	1.2 (0.8–2.0)

Of the 31225 dispensed prescriptions, 1.0% (312/31225) necessitated a clarification contact with the prescriber. These comprised 1.6% (226/14365) of all new prescriptions and 0.5% (86/16860) of all refill prescriptions. This yielded an RR of 3.1 (95% CI 2.4–4.0) for new prescriptions compared to refill prescriptions (Table 2).

The prescribers accepted 89.5% of the suggested pharmacist interventions, 77.7% (192/247) as suggested and an additional 11.7% (29/247) after a modification by the prescriber during the contact (Table 3). Each prescription necessitating a contact with a prescriber contains one or more interventions.

**Table 3:** Results of suggested interventions per type of prescription.

Type of clarification contact	All prescriptions		Refill prescriptions		New ePrescriptions		New non-electronic prescriptions	
Number	(N = 312)	%	(N = 86)	%	(N = 147)	%	(N = 79)	%
Contact with prescriber	247	79.2	64	74.4	123	83.7	60	75.9
- suggestion accepted	192	61.5	50	58.1	91	61.9	51	64.6
- suggestion accepted with modification	29	9.3	10	11.6	14	9.5	5	6.3
- suggestion not accepted	14	4.5	1	1.2	11	7.5	2	2.5
- other	10	3.2	2	2.3	7	4.8	1	1.3
Missing value	2	0.6	1	1.2	-	-	1	1.3
Unsuccessful attempt to contact the prescriber	65	20.8	22	25.6	24	16.3	19	24.1

When new and refill prescriptions were pooled together, the median recorded duration of a contact with a prescriber was lower for ePrescriptions (4 minutes) compared to non-ePrescriptions (5 minutes).

## Discussion

New ePrescriptions were associated with clarification contacts to a greater extent than new non-electronic prescriptions. This was mainly due to missing or ambiguous information for 'Dosage and directions for use'. This may be due to the widespread use of abbreviations, such as '1t3d' (one tablet three times daily) which have not been standardized among different EHRs. The abbreviations may be translated by the EHRs in such a way that was unanticipated by the prescribers prior to transferring the prescription to the pharmacy. As only one in a hundred prescriptions necessitated a prescriber contact, ePrescriptions might still be more efficient and cost-effective overall compared to non-electronic prescriptions. However, if the potential of ePrescriptions is to be fulfilled, quality aspects must be more pronounced in future deployment.

ePrescribing in Sweden, has been subject to a low degree of governmental regulation. Nevertheless, the technical standards have been mutually agreed by all actors on a national level. A need for more detailed standards has been recognized, resulting in the implementation of a standardized new ePrescription format (NEF). Also, a national working party for modernization of ePrescribing (MER) is developing next-generation applications. Some of the weaknesses in the ePrescribing process detected in the present study are expected to be improved with these new standards. The introduction of an extensive electronic process in health care involves a variety of different actors, emphasizing the need for a national infrastructure for continuous change management, including strategies, policies, and implementation for improved quality and safety.

The majority of the pharmacists' clarification contacts were accepted by the prescribers, which is in accordance with other studies [28]. This result emphasizes the benefits of pharmacists as gatekeepers. It also underlines the ongoing need for pharmacists to act in this area in order to prevent the patient from incurring prescription errors. If undiscovered, such errors may result in serious adverse drug reactions and even hospitalization [29,30]. On the other hand, there is an obvious benefit of designing the different EHRs to minimize primary errors. Such a strategy would also reduce the need for pharmacist intervention.

### Limitations

In our study clarification contacts were detected for 0.5–1.6% of the prescriptions. The proportion of prescription errors seen in other studies varies between 0.9% and 8.7%. The largest value was detected at a mail-order pharmacy [23,31-35]. The reasons for this wide range of percentage values may include differences in definitions, methodology, culture, legislation and the technical solutions available. One major reason for the rather low frequency in our study compared to other studies may be that we only included prescribing errors that the dispensing pharmacists considered severe enough to necessitate a clarification contact with the prescriber. For example, the 'dose and directions for use' texts were edited by pharmacists for readability and correctness to a much greater extent than reported here.

One reason for the observed increased RR for ePrescriptions compared to paper prescriptions could be that prescribers who adopt new technology at an early phase differ from their more cautious colleagues in terms of carefulness and accuracy. The increased need for clarification contacts with prescribers may also reflect a need for more user training in the new technology.

The reported error rates in other studies employing self-reported interventions have shown tenfold variation which may indicate low validity. To ascertain a high degree of capture we used independent observers to document the interventions. The alignment of the work organization and the supervision of the protocol employed were chosen in order to achieve consistent reporting which minimized the inter-individual variation. Nevertheless, in observational studies the risk of misclassification must be taken into account. We are of the opinion that our results were not influenced to any considerable extent by misclassification in the data gathered specifically for the study. However, we cannot rule out the risk of misclassification in the statistical data acquired from Apoteket AB. We made an attempt to evaluate the risk of misclassification in a small sample (1 pharmacy, 2 days), and found that in some cases refill prescriptions (< 5%) could have been recorded as new prescriptions. A sensitivity analysis revealed that our risk estimate for ePrescriptions, RR 1.7, could be a slight overestimate. An assumed differential misclassification of 5% of the non-electronic prescriptions yielded an RR of 1.5 (95% CI 1.1–1.9).

### Improvement of quality and safety

Medical errors constitute one of the most important quality problems in health care today [36]. Studies have shown that 2.4–7% of admissions to inpatient facilities were caused by adverse drug reactions [37]. Of these, 50% were deemed to be preventable [36]. Prescribing errors comprise one important contributable cause to adverse drug reactions. The reason for these unintended errors might be due to imperfect user interface and design of the ePrescribing computer systems.

The introduction of computerized prescribing was expected to increase quality and safety for patients in health care. Nevertheless, computerized order entry systems (CPOE) in hospitals have been shown to actually facilitate medication errors [20]. The unintended consequences of CPOE are widespread and can be both positive and negative, and continue to exist over the duration of use. Aggressive detection and management of adverse unintended consequences is considered vital for CPOE success [38]. By identifying and understanding the types and causes of unintended adverse consequences associated with CPOE, system developers and implementers are expected to better manage implementation and maintenance of CPOE [39].

Increasingly, the prescription information entered by the prescriber into the EHR will not be used solely for pharmacy dispensing of prescriptions, but also by the patients for medication lists and online medication history [40-42]. The information will also be used for clinical decision making by *other *physicians and for epidemiological research and evaluation. This underscores the need for continuous monitoring of quality and improvement [22].

### Future aspects

With the majority of prescriptions transferred as ePrescriptions, there is an obvious need for improved evaluation and certification of the information and communication technology (ICT) systems which produce drug prescriptions. Our study has highlighted a number of aspects of ePrescribing in need of improvement. These aspects should be followed up in subsequent studies. ICT systems for ePrescribing must also be evaluated intermittently with compulsory certification to assure patients of high quality health care [21,43].

## Conclusion

The increased proportion of prescriptions necessitating a clarification contact for new ePrescriptions, compared to new non-electronic prescriptions, indicates the need for an increased focus on quality aspects in ePrescribing deployment. ETP technology should be developed towards a two-way communication between the prescriber and the pharmacist with automated checks for missing, inaccurate, or ambiguous information. Such a process would increase safety and quality for the patient and improve efficiency and cost-effectiveness within the health care system.

## Competing interests

At the time of the study, two of the authors (BÅ, AE) were employed by Apoteket AB, a corporation fully owned by the Swedish government to run all Swedish pharmacies, including MOPs. The eHealth institute, University of Kalmar is partly funded by Apoteket AB. Apoteket AB is also obliged by the Swedish government to provide sales statistics for the Swedish pharmaceutical market.

## Authors' contributions

BÅ was responsible for the analysis and the draft of the manuscript. EM carried out the data analysis and participated in the drafting of the manuscript. GP participated in the analysis and the revision of the manuscript. AE conceived the study, participated in the design and coordination of the study and supervised the draft of the manuscript. All authors read and approved the final manuscript.

## Pre-publication history

The pre-publication history for this paper can be accessed here:

http://www.biomedcentral.com/1472-6947/9/8/prepub

## Supplementary Material

Additional file 1 Protocol form translated to English Click here for file
