# Supplementary figures and images for: Assessment of ePrescription quality: an observational study at three mail-order pharmacies
Source: BMC Med Inform Decis Mak. 2009 Jan 26;9:8. doi: 10.1186/1472-6947-9-8 (PMC2654447; doi:10.1186/1472-6947-9-8)

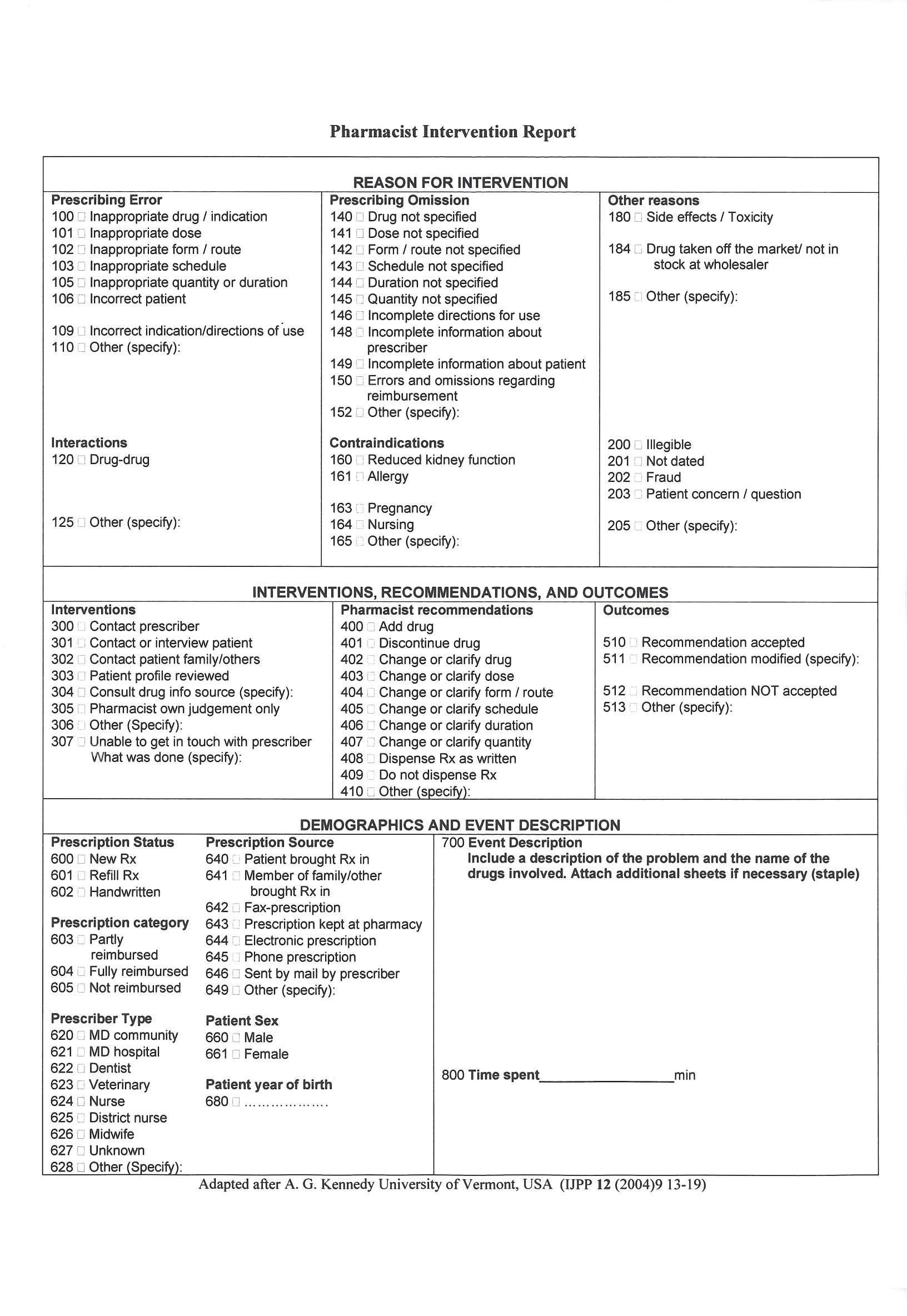

Supplement: Additional file 1 — Protocol form translated to English [file 1472-6947-9-8-S1.doc]
